# A comparison between angiotensin converting enzyme inhibitors and angiotensin receptor blockers on end stage renal disease and major adverse cardiovascular events in diabetic patients: a population-based dynamic cohort study in Taiwan

**DOI:** 10.1186/s12933-016-0365-x

**Published:** 2016-04-02

**Authors:** Lung-Sheng Wu, Shang-Hung Chang, Gwo-Jyh Chang, Jia-Rou Liu, Yi-Hsin Chan, Hsin-Fu Lee, Ming-Shien Wen, Wei-Jan Chen, Yung-Hsin Yeh, Chi-Tai Kuo, Lai-Chu See

**Affiliations:** Division of Cardiology, Department of Internal Medicine, Chang Gung Memorial Hospital, Linkou and Chang Gung University College of Medicine, Linkou, Tao-Yuan Taiwan; Graduate Institute of Clinical Medicinal Sciences, College of Medicine, Chang Gung University, Tao-Yuan, Taiwan; Department of Public Health, College of Medicine, and Biostatistics Core Laboratory, Molecular Medicine Research Center, Chang Gung University, Tao-Yuan, Taiwan; Division of Rheumationalogy and Allergy and Immunology, Department of Internal Medicine, Chang Gung Memorial Hospital, Linkou, Taiwan

**Keywords:** Angiotensin converting enzyme inhibitors, Angiotensin receptor blockers, Diabetes mellitus, Major adverse cardiovascular events, End-stage renal disease

## Abstract

**Background:**

Contemporary guidelines recommend angiotensin-converting-enzyme inhibitors (ACEi) or angiotensin-receptor blockers (ARB) for hypertensive patients with diabetes. However, there is limited data to evaluate the comparison between ACEi and ARB on end stage renal disease (ESRD) and major adverse cardiovascular events (MACE), in Asian diabetic patients.

**Methods:**

We used the Taiwan Longitudinal Cohort of Diabetes Patients Database to perform a population-based dynamic cohort study. The comparison between ACEi and ARB on ESRD and MACE in diabetic patients was examined using the propensity score weighting method. We followed these patients until the occurrence of first study outcomes or end date of the study, whichever came first.

**Results:**

There were 6898 and 12,758 patients in ACEi and ARB groups, respectively. The mean follow-up period was about 3.5 years in ESRD and 2.5 years in MACE. The incidence of ESRD was 0.44 % and 0.63 % per person-years in the ACEi and ARB group, respectively. The risk of ESRD was lower in the ACEi group than the ARB group [hazard ratio (HR) 0.69; 95 % confidence interval (CI) 0.54–0.88, P = 0.0025]. Among those without chronic kidney disease (CKD), the incidence of ESRD was 0.30 % and 0.37 % per person-years in the ACEi and ARB group, respectively. ACEi was similar to ARB in preventing ESRD for those without CKD (P = 0.11). Among those with CKD, the incidence of ESRD was 1.39 % and 2.34 % per person-years in the ACEi and ARB group, respectively. The ACEi group had a lower risk of ESRD than the ARB group (HR 0.61; 95 % CI 0.42–0.88, P = 0.008). The incidence of MACE was 9.33 % and 9.62 % per person-years in the ACEi and ARB group, respectively. There was no significant difference in the composite MACE outcome between the two groups (P = 0.42), but the ACEi group was associated with a higher risk of stroke than the ARB group (HR 1.12; 95 % CI 1.02–1.24, P = 0.02).

**Conclusions:**

ACEi compared with ARB was associated with a lower incidence of ESRD, especially in those with CKD. Though ACEi and ARB had a similar risk of composite MACE outcome, ACEi had a slightly higher incidence of stroke than ARB, among the Asian diabetic patients.

**Electronic supplementary material:**

The online version of this article (doi:10.1186/s12933-016-0365-x) contains supplementary material, which is available to authorized users.

## Background

Diabetes mellitus (DM) is a global health problem and a major cause of cardiovascular disease and end-stage renal disease (ESRD) [1, 2]. According to current guidelines [1, 3], angiotensin-converting enzyme inhibitors (ACEi) and angiotensin receptor blockers (ARB) are recommended for the treatment of hypertension, and especially for diabetic patients. Both drugs block angiotensin II, however ACEi are characterized by a decrease in the degradation of bradykinin leading to a release of nitric oxide and prostaglandins resulting in additional vasodilatation. The differences in the modes of action between ACEi and ARB may have clinical implications for diabetic patients [4, 5]. A recently published meta-analysis [6] showed that ACEi were associated with a significant reduction (14 %) in major cardiovascular events, but that ARB had no benefit on these outcomes. In these patients, management of cardiovascular risk factors and careful monitoring eGFR may represent opportunities to reduce the risk of major adverse cardiovascular events (MACE) [7]. In addition, these two agents have been reported to be beneficial in delaying the progression of kidney disease in patients with non-dialysis-dependent chronic kidney disease (CKD) [8–10]. However, studies comparing ACEi and ARB with regards to renoprotective effects are limited.

Cough occurs in 5–20 % of patients treated with ACEi. The mechanism may involve accumulation of prostaglandins, kinins, or substance P, as both bradykinin and substance P are degraded by ACE [11]. Furthermore, the incidence of discontinuing ACEi due to cough has been reported to be up to 30 % in Asian patients [12, 13]. Therefore, it is important to have a comparison of the efficacy of these two classes of drugs in Asian diabetic patients. Given the uncertainty surrounding the effect of ACEi/ARB, we performed a retrospective, population-based dynamic cohort study to compare the efficacy between ACEi and ARB in preventing ERSD and MACE in diabetic patients.

## Research design and methods

### Study population

The National Health Insurance (NHI) program, a compulsory universal health care system in Taiwan, was launched in 1995 and currently covers 23.72 million enrollees or about 99 % of the population. The National Health Research Institute (NHRI) is responsible for managing and maintaining all insurance claims data, and various datasets are released for research purposes after thorough review and after all personal information has been encrypted. Hence, informed consent was not required for this study. The study protocol conformed to the ethical guidelines of the 1975 Declaration of Helsinki and was approval by the Institutional Review Board of the Chang Gung Medical Foundation, Taiwan (103-7871C).

**Fig. 1:**
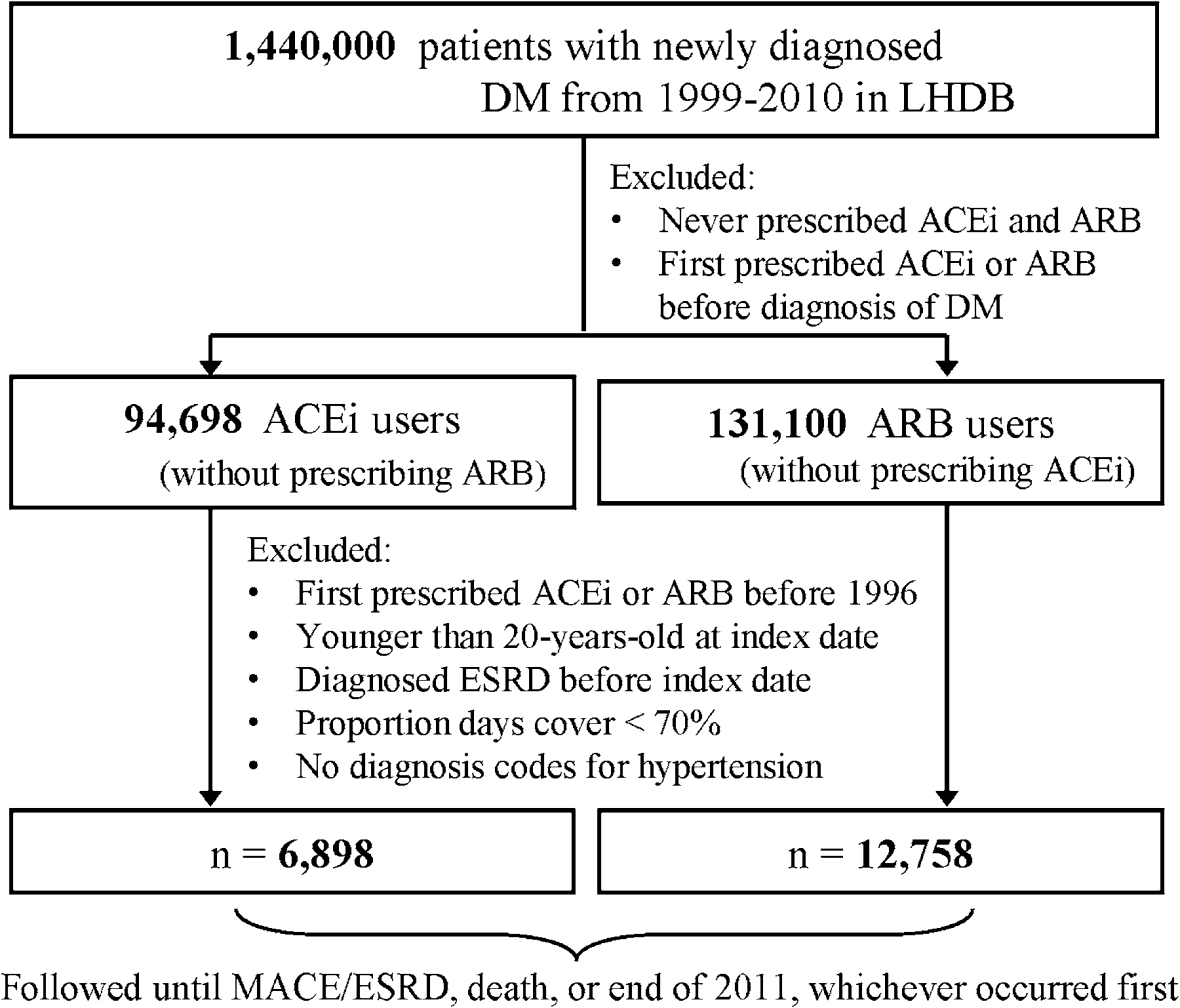
Flowchart of subject selection. *LHDB* longitudinal cohort of diabetes patients, *DM* diabetes mellitus, *ACEi* angiotensin converting enzyme inhibitors, *ARB* angiotensin receptor blockers, *ESRD* end stage renal disease, *MACE* major adverse cardiovascular events

Data from the Longitudinal Cohort of Diabetes Patients (LHDB) released by the NHRI were used for this study. In LHDB, patients who were first diagnosed with DM from 1999 to 2010 were eligible for this study, if they: (1) had at least one hospitalization with DM as one of the discharge codes (A-code A181 before 2000 or ICD-9-CM code 250 after 2000) or used DM medications (Additional file 1); (2) had at least two outpatient visits due to DM in the same year; (3) had no hospitalizations or outpatient visits due to DM from 1996 to 1998; (4) were over 20 years of age. Due to the restriction set by the NHRI of releasing data no more than 10 % of the total population, an annual total of 120,000 eligible patients from 1999 to 2010 were randomly selected, and their original claims data including admissions, outpatient visits, and medications were recorded in the LHDB. The LHDB included data before a diagnosis of DM and treatment for patients with and without DM. The LHDB has been tested and confirmed by other studies to be representative of Taiwanese patients with DM [14–18]. In this study, ACEi and ARB were listed in the supplement. Patients in LHDB who had been prescribed ACEi or ARB before the first diagnosis of DM, or who had been prescribed neither ACEi nor ARB were excluded. ACEi users were those who took ACEi but not ARB. The ARB group were those who took ARB but not ACEi. The index date was defined as the calendar date of a first prescription of ACEi or ARB. Our selection further excluded if (1) the year of their first prescription was before 1996, or (2) they were younger than age 20 years of age on index date, or (3) they had ESRD before index date, or (4) they did not had hypertension before index date, or (5) cover rate less than 70 %. The follow-up period was from the index date until either the date of new onset of MACE or ESRD (when treated as an outcome variable), or 31 December 2011, whichever came first (Fig. 1). Note that the nature of LHDB and this study design is qualified as a dynamic cohort, in which eligible participants were recruited when newly diagnosed patients appeared during the study. The advantage of a dynamic cohort is that the number of participants does not decline over time, and that aging of the study participants over time does not weaken the study.

A patient is considered to have reached the end points of the study, the occurrence of MACE, if he or she has bee diagnosed with one of the codes: International Classification of Diseases, Ninth Revision, Clinical Modification (ICD-9-CM) codes as follows: myocardial infarction (410), congestive heart failure (428), stroke (430–437), malignant dysrhythmia (426.0, 426.12–426.13, 426.51–426.52, 426.54, 427.1, 427.4, 427.41–427.42, 427.5), cardiogenic shock (785.51); or procedure codes of the Taiwan NHI for percutaneous coronary intervention (PCI) (33076A, 33076B, 33077A, 33077B, 33078A, 33078B), coronary artery bypass surgery (CABG) (68023A, 68023B, 68024A, 68024B, 68025A, 68025B), and thrombolysis therapy (B016526248, K000743248, K000744238) [19]. A previous MACE is defined as a hospitalization due to MACE before the index date. A new MACE is defined as a hospitalization with MACE as the primary diagnosis 14 days after the index date.

Patients with chronic kidney disease are defined as those who were diagnosed with ICD9-CM codes 580–589 at least twice at an outpatient clinic or a discharge. Patients with hyperlipidemia are defined as those who were diagnosed with ICD9-CM code 272 or A182 at least twice at an outpatient clinic or a discharge.

Patients with ESRD requiring chronic renal replacement therapy are eligible for a catastrophic illness certificate in Taiwan. Patients with a catastrophic illness certificate are entitled to a waiver for medical co-payment. Diagnostic information is sent to the insurance administration for a review by a panel of commissioned experts to review the diagnosis and approve the waiver. Consequently, ESRD is defined as patients with a catastrophic illness certificate for ESRD (ICD-9-CM code: 585).

### Statistical analysis

We use the propensity score method to compare between ACEi and ARB on the ESRD and MACE in DM patients to eliminate the effects of unbalanced demographic and comorbid medical disorders at index date for observational data. The propensity score was the predicted probability of being in ACEi group derived from the fitted logistic regression, in which group status was regressed on covariates at index date (Table 1). Inverse probability of treatment weights (IPTWs) using the propensity scores was then applied to balance covariates across the two study groups. The standardized mean difference (SMD) rather than using statistical testing was made to examine the balance of covariates at index date between the two study groups, because balance is a property of the sample and not of an underlying population. The absolute value of SMD ≤ 0.1 indicates a negligible difference in covariates between the two study groups [20]. In time-to-event analyses, incident rate, crude hazard ratio (log-rank test) and adjusted hazard ratio (Cox’s proportional hazard model) were estimated, accounting for the weighted nature of two study groups with robust variance estimation [21]. Statistical significance was defined as a P value less than 0.05. All statistical analyses were performed using SAS 9.2 (SAS Institute Inc., Cary, NC, USA).

**Table 1:** The demographic and comorbid medical disorders at index date, before and after propensity score weighting, among hypertensive and diabetic patients who were prescribed with either angiotensin-converting enzyme inhibitors (ACEi) or angiotensin receptor blockers (ARB)

	Propensity score weighting					
	Before			After		
	ACEi (n = 6898)	ARB (n = 12,758)	Standardized mean difference	ACEi (n = 6898)	ARB (n = 12,758)	Standardized mean difference
Duration from DM to index date^a^ (years)	3.07 ± 3.15	3.69 ± 3.33	−0.1931	3.17 ± 3.15	3.08 ± 2.26	−0.0032
Age (years) *(mean* *±* *SD)*	61.5 ± 13.0	62.4 ± 12.8	−0.0711	61.5 ± 13.0	61.3 ± 9.7	0.0183
Age group			0.0944			0.0053
20–49 (%)	20.9	17.2		20.9	21.0	
50–64 (%)	39.4	40.3		39.4	39.5	
65+ (%)	39.7	42.4		39.7	39.5	
Male sex	59.5 %	53.1 %	0.1271	59.5 %	59.5 %	−0.0016
Congestive heart failure	4.51 %	4.85 %	−0.0163	4.51 %	4.51 %	−0.0000
Stroke	12.73 %	14.70 %	−0.0572	12.73 %	12.73 %	−0.0000
Malignant dysrhythmia	0.45 %	0.49 %	−0.0054	0.45 %	0.45 %	0.0004
Cardiogenic shock	0.32 %	0.23 %	0.0176	0.32 %	0.33 %	−0.0025
MI/PCI	5.00 %	4.56 %	0.0206	5.00 %	4.98 %	0.0010
CABG	0.46 %	0.60 %	−0.0192	0.46 %	0.46 %	0.0012
Thrombolysis therapy	0.25 %	0.24 %	0.0023	0.25 %	0.24 %	0.0018
Hyperlipidemia	54.54 %	59.81 %	−0.1066	54.54 %	54.68 %	−0.0029
Chronic kidney disease	13.51 %	16.73 %	−0.0901	13.51 %	13.51 %	0.0001

*DM* diabetes mellitus, *ACEi* angiotensin converting enzyme inhibitors, *ARB* angiotensin receptor blockers, *MI* myocardial infarction, *PCI* percutaneous coronary intevention, *CABG* coronary artery bypass graft surgery

^a^Index date was the date first prescribed either ACEi or ARB

## Results

The demographic characteristics and comorbid medical disorders at the index date are shown in Table 1. There were 6898 patients in ACEi and 12,758 patients in ARB groups, respectively. On average, patients were prescribed either ACEi or ARB 3 years after being diagnosed with DM. The mean age at the first prescription of either ACEi or ARB was 61.5 ± 13.0 and 62.4 ± 12.8 years, respectively. Approximately half (54.5 %) had hyperlipidemia, 13.5 % had CKD, 12.7 % had stroke and 5 % had myocardial infarction (MI) or PCI. A history of congestive heart failure, cardiogenic shock, CABG, and thrombolysis therapy were not common (from 0.25 to 4.51 %). After propensity score weighting, the age, gender and comorbid medical disorders were comparable with absolute values of standardized mean difference <0.1 between the two study groups.

**Fig. 2:**
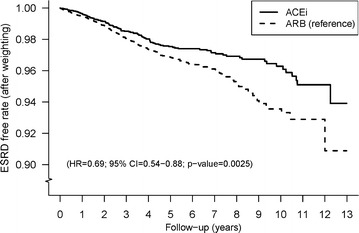
Overall end stage renal disease free rates for ACEi and ARB users after propensity score weighting. *ACEi* angiotensin converting enzyme inhibitors, *ARB* angiotensin receptor blockers, *ESRD* end stage renal disease

During 26,809 person-years with a mean follow-up of 3.9 years in the ACEi group and 41,292 person-years with a mean follow-up of 3.2 years in the ARB group, the incidence (person-years) of ESRD was 0.44 % in the ACEi group and 0.63 % in the ARB group (Table 2). Figure 2 displays the Kaplan–Meier curves of ESRD-free rate among the ACEi and ARB group. The ACEi group had a lower incidence of ESRD than the ARB group (HR 0.69; 95 % CI 0.54–0.88, P = 0.0025).

**Table 2:** Incidence (per 100 person-years) of outcomes among the ACEi and ARB users after propensity score weighting

	Incidence (95 % CI)		Hazard ratio (95 % CI)	
	ACEi (n = 6898)	ARB (n = 12,758)	ACEi vs. ARB	*P* value
MACE				
Any MACE^a^	9.33 (8.90−9.76)	9.62 (9.16−10.08)	1.03 (0.96−1.10)	0.4244
Cogestive heart failure	1.48 (1.33−1.63)	1.51 (1.35−1.67)	1.06 (0.91−1.22)	0.4538
Stroke	3.78 (3.53−4.03)	3.56 (3.31−3.82)	1.12 (1.02−1.24)	0.0230
Malignant dysrhythmia	0.16 (0.12−0.21)	0.15 (0.10−0.20)	1.14 (0.73−1.77)	0.5571
Cardiogenic shock	0.03 (0.01−0.06)	0.04 (0.02−0.07)	0.81 (0.31−2.07)	0.6547
MI or PCI	1.77 (1.61−1.93)	2.19 (2.00−2.39)	0.89 (0.78−1.01)	0.0646
CABG	0.18 (0.13−0.23)	0.22 (0.16−0.28)	0.89 (0.60−1.31)	0.5438
Thrombolysis therapy	0.00 (0.00−0.02)	0.02 (0.01−0.05)	0.19 (0.02−1.66)	0.1339
ESRD	0.44 (0.36−0.52)	0.63 (0.53−0.73)	0.69 (0.54−0.88)	0.0025

*ACEi* angiotensin converting enzyme inhibitors, *ARB* angiotensin receptor blockers, *CI* confidence interval, *MACE* major adverse cardiovascular disease, *MI* myocardial infarction, *CHF* cogestive heart failure, *PCI* percutaneous coronary intevention, *CABG* coronary artery bypass graft surgery, *ESRD* end stage renal disease

^a^MACE = CHF or stroke or malignant dysrhythmia or cardiogenic shock or MI or PCI or CABG or thrombolysis therapy

**Fig. 3:**
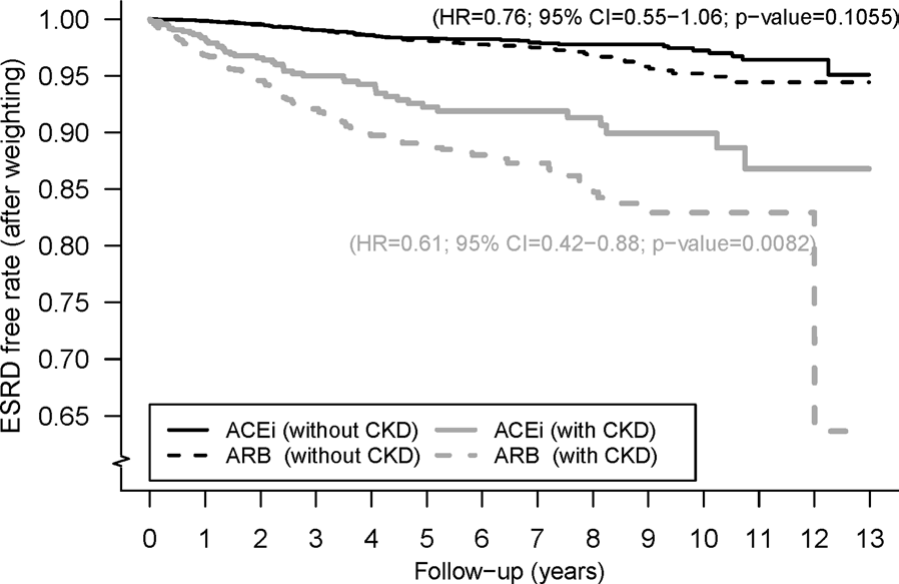
End stage renal disease free rates stratified by chronic kidney disease or not for ACEi and ARB users after propensity score weighting. *ACEi* angiotensin converting enzyme inhibitors, *ARB* angiotensin receptor blockers, *ESRD* end stage renal disease, *CKD* chronic kidney disease

Furthermore, we evaluated the effect of CKD on the incidence of ESRD. There were 5966 and 10,623 patients without CKD in ACEi and ARB group, respectively. Among those without CKD, the mean age at the first prescription of either ACEi or ARB was 60.8 and 60.6 years, respectively. Approximately half (53.8 %) had hyperlipidemia, 12.2 % had stroke and 5 % had MI or PCI. A history of congestive heart failure, cardiogenic shock, CABG and thrombolysis therapy were not common (from 0.3 to 4.0 %). During 23,353 person-years with a mean follow-up of 3.9 years in ACEi group and 34,928 person-years with a mean follow-up of 3.3 years in ARB group, the incidence (person-years) of ESRD was 0.30 and 0.37 % in the ACEi and ARB group, respectively (Table 3). There were no significant differences in ESRD between the two groups (HR 0.76; 95 % CI 0.55–1.06, P = 0.11) (Fig. 3).

**Table 3:** The demographic and comorbid medical disorders at index date after propensity score weighting among hypertensive and diabetic patients who were prescribed with either angiotensin-converting enzyme inhibitors (ACEi) or angiotensin receptor blockers (ARB) stratified by chronic kidney disease or not

	Propensity score weighting					
	With CKD			Without CKD		
	ACEi (n = 932)	ARB (n = 2135)	Standardized mean difference	ACEi (n = 5966)	ARB (n = 10,623)	Standardized mean difference
Duration from DM to index date (years)	3.50 ± 3.33	3.52 ± 2.09	−0.0049	3.00 ± 3.11	3.01 ± 2.29	−0.0035
Age (years) *(mean* *±* *SD)*	65.5 ± 13.3	65.2 ± 8.8	0.0276	60.8 ± 12.9	60.6 ± 9.8	0.0175
Age group (years)			0.0123			0.0049
20–49 (%)	14.70	15.06		21.81	21.92	
50–64 (%)	32.08	32.25		40.56	40.69	
65+ (%)	53.22	52.69		37.63	37.39	
Male sex	59.12 %	59.21 %	−0.0019	59.52 %	59.62 %	−0.0019
Congestive heart failure	8.05 %	8.15 %	−0.0039	3.96 %	3.94 %	0.0006
Stroke	16.20 %	16.15 %	0.0014	12.19 %	12.16 %	0.0007
Malignant dysrhythmia	0.21 %	0.21 %	0.0014	0.49 %	0.48 %	0.0011
Cardiogenic shock	0.64 %	0.73 %	−0.0102	0.27 %	0.27 %	−0.0011
MI/PCI	5.15 %	5.08 %	0.0030	4.98 %	4.96 %	0.0008
CABG	0.11 %	0.11 %	−0.0010	0.52 %	0.51 %	0.0008
Thrombolysis therapy	0.00 %	0.00 %	−0.0002	0.28 %	0.28 %	0.0012
Hyperlipidemia	59.44 %	59.86 %	−0.0085	53.77 %	53.77 %	−0.0017
Incidence of ESRD^a^	1.39	2.34		0.30	0.37	
(95 % CI)	(1.00−1.78)	(1.80−2.89)		(0.23−0.37)	(0.29−0.46)	

*DM* diabetes mellitus, *ACEi* angiotensin converting enzyme inhibitors, *ARB* angiotensin receptor blockers, *CKD* chronic kidney diseases, *MI* myocardial infarction, *PCI* percutaneous coronary intevention, *CABG* coronary artery bypass graft surgery

^a^Incidence per 100 person-years

There were 932 and 2135 patients with CKD in the ACEi and ARB group, respectively. The mean age was 65.5 years in the ACEi group and 65.2 years in the ARB group. Approximately half (59 %) had hyperlipidemia, 16.2 % had stroke, 8.1 % had CHF and 5.2 % had MI or PCI. A history of cardiogenic shock, CABG and thrombolysis therapy were not common (from 0 to 0.7 %). Among those with CKD, during 3457 person-years with a mean follow-up of 3.7 years in the ACEi group and 6364 person-years with a mean follow-up of 3.0 years in the ARB group, the incidence rate (person-years) of ESRD was 1.39 and 2.34 % in the ACEi and ARB group, respectively (Table 3). The ACEi group had a lower rate of the ESRD than with ARB group (HR 0.61; 95 % CI 0.42–0.88, P = 0.008) (Fig. 3).

**Fig. 4:**
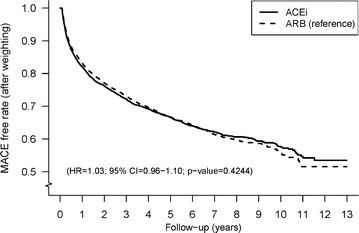
Overall major adverse-cardiovascular-events free rates for ACEi and ARB users after propensity score weighting. *ACEi* angiotensin converting enzyme inhibitors, *ARB* angiotensin receptor blockers, *MACE* major adverse cardiovascular events

**Fig. 5:**
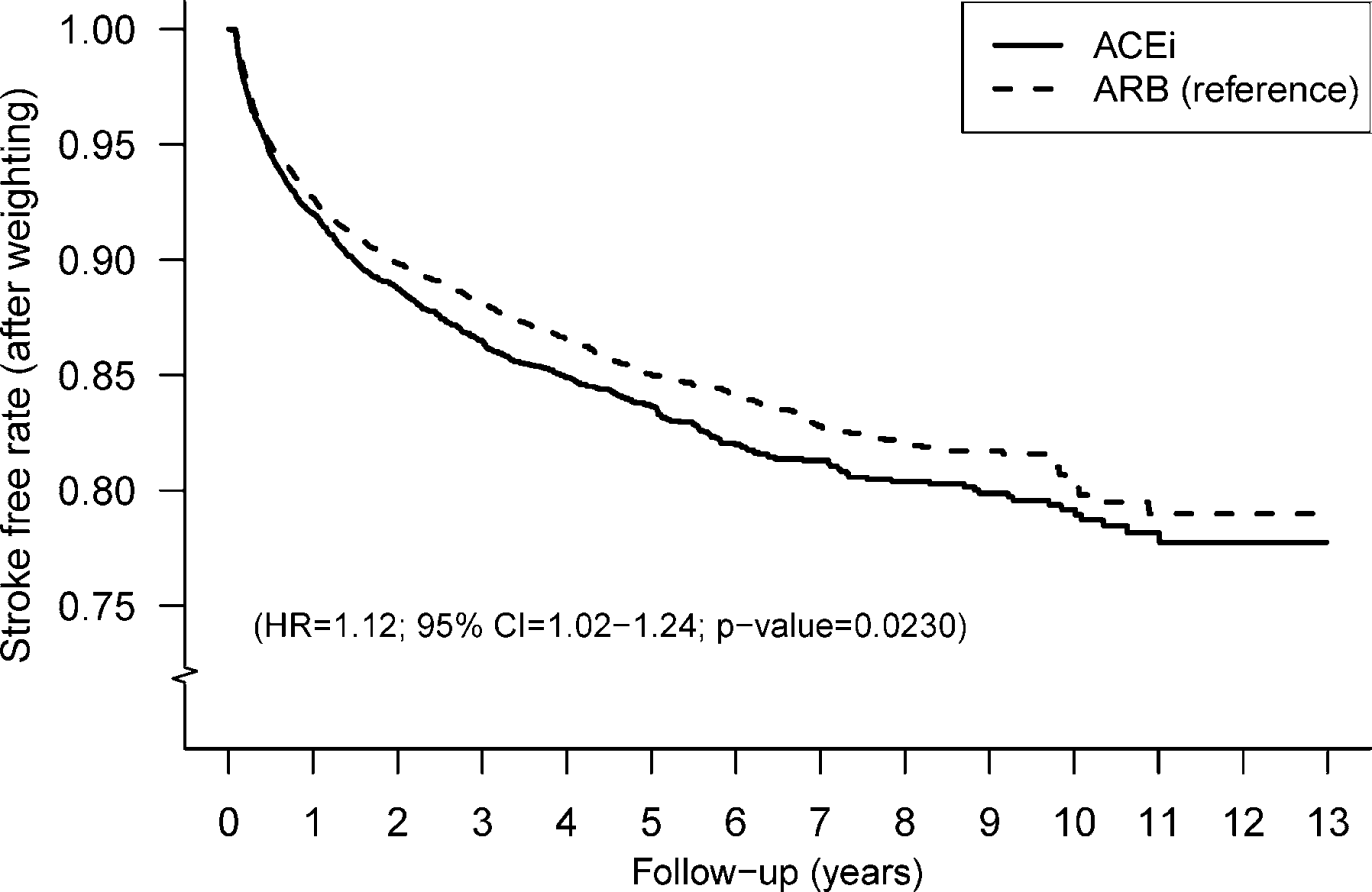
Overall stroke free rates for ACEi and ARB users after propensity score weighting. *ACEi* angiotensin converting enzyme inhibitors, *ARB* angiotensin receptor blockers

During 19,203 person-years with a mean follow-up of 2.8 years in the ACEi group and 30,389 person-years with a mean follow-up of 2.4 years in the ARB group, the incidence rate (person-years) of any MACE was 9.33 and 9.62 % in the ACEi and ARB groups, respectively (Table 2). Figure 4 displays the Kaplan–Meier curves of MACE free rate for the two study groups. There were no significant differences in MACE between the two groups (HR 1.03; 95 % CI 0.96–1.10, P = 0.42). Stroke, MI or PCI and congestive heart failure were the most common MACE in both the ACEi and ARB groups, with incidence (person-years) of 3.78/3.56, 1.77/2.19, and 1.48/1.51 %, respectively. Note that the ACEi group had a higher incidence of stroke than the ARB group (HR 1.12; 95 % CI 1.02–1.24, P = 0.02) (Table 2). Figure 5 displays the Kaplan–Meier curves of stroke- free rate among the ACEi and ARB group.

## Discussion

In this study, we compared the long-term impact of ACEi and ARB in Asian diabetic patients, using a population-based dynamic cohort study design and propensity score weighting method. Our results demonstrated that ACEi compared with ARB was associated with a lower incidence of the progression to ESRD in diabetic patients, especially in the patients with CKD. In addition, though ACEi and ARB had a similar incidence of the composite MACE outcome, ARB had a lower incidence of the stroke than ACEi.

Clinical studies of ACEi and ARB have shown that they reduce albuminuria and the loss of glomerular filtration rate and the need for dialysis in those with advanced renal disease [22]. Data from the ONTARGET trial [23], which focused on diabetic patients showed that the effect of telmisartan on dialysis was similar to ramipril with rates of hemodialysis of 0.6 and 0.56 %, respectively (P = 0.747). A retrospective cohort study, using Veterans Affairs databases showed that ACEIs are associated with lower ESRD development in diabetic patients compared with ARB (Odds ratio 0.10; 95 % CI 0.04–0.21, P = 0.02) [24]. In this study, focused on diabetic patients with hypertension, we demonstrated that ACEi was superior to ARB in preventing ESRD development (HR 0.69; 95 % CI 0.54–0.88, P = 0.0025). Lacourciere et al. demonstrated that among diabetic patients with normal glomerular filtration rate (GFR), the rate of decline in GFR with 1 year follow up is similar between patients taking either ACEi or ARB [25]. Our study with longer follow-up duration (mean 3.5 years) also showed the ACEi/ARB have similar effect in progression to ESRD among the diabetic patients without CKD. In addition, among the patients with CKD, our study demonstrated that ACEi was associated with a lower risk of ESRD than ARB (HR 0.61, 95 % CI 0.42–0.88, P = 0.008). We saw that ACEi with a better renoprotective effect than ARB, primary from these with CKD. There are two important differences between ACEi and ARB (angiotensin II type I antagonists). The first is the blockade of bradykinin degradation by ACEi and the second is the supraphysiologic activation of the Angiotensin II type 2 receptor (AT2) when ARB (AT1 antagonists) are administered [26]. AT2 receptor plays a counterregulatory protective role mediated via bradykinin and nitric oxide against the antinatriuretic and pressor actions of angiotensin II [27]. These mechanisms may explain why ACEi has better renoprotection than ARB. Some studies have suggested that dual blockage of renin–angiotensin system may provide additive benefit in diabetic nephropathy, but further studies are needed to validate such therapy, which should be used with caution [28].

In our study, the incident rates of cardiovascular comorbidities including myocardial infarction, congestive heart failure, PCI and stroke were relatively lower, as compared with the HOPE [29], TRANSCEND [30], or ONTARGET [31] studies. The ONTARGET study (Asian population 13.8 %, 1200 patients) compared the ACEi (ramipril) and the ARB (telmisartan) in patients with vascular disease or high-risk diabetes (37 %), and found that telmisartan was equivalent to ramipril in reducing mortality and morbidity from cardiovascular causes. We also found that treatment with ACEi and ARB had a similar incidence of the composite MACE outcome in Asian diabetic patients. On the other hand, we found that among diabetic patients, ACEi has a slightly higher risk of stroke than ARB (HR 1.12; 95 % CI 1.02–1.24, P = 0.023). In other words, our study demonstrated that ARB has a slightly better protective effect on stroke than ACEi among the diabetic patients. A meta-analysis [32], which did not focus on the diabetic patients, also showed that ARB was more protective than ACEi on the risk of stroke (odds ratio 0.92; 95 % CI:0.85–0.99, P = 0.037).

There are several limitations to this study. First, nearly all hospitals/clinics are linked to the National Health Insurance (NHI) program, which covered over 99 % of the population covered by the program during the study period. However, it is possible that we were not able to assess some cases of MACE. Second, several important clinical parameters are not available in the NHI claims database, including clinical/imaging information, severity of MACE, smoking history, albuminuria, LDL-cholesterol, and creatinine level. Third, the benefit of antiplatelet agents in primary prevention for cardiovascular events is still under debate, so we do not examine the effect of antiplatelet agents in MACE and ESRD. [33].

In conclusion, among Asian diabetic patients, ACEi appeared to have a lower risk of ESRD development than ARB, especially in those with chronic kidney disease. Despite the similar incidence of the composite MACE outcome, ARB was slightly more protective than ACEi on the risk of stroke.

## Additional file

10.1186/s12933-016-0365-x The drugs analyzed are listed as following.
