# Supplementary material for: A comparison between angiotensin converting enzyme inhibitors and angiotensin receptor blockers on end stage renal disease and major adverse cardiovascular events in diabetic patients: a population-based dynamic cohort study in Taiwan
Source: Cardiovasc Diabetol. 2016 Apr 2;15:56. doi: 10.1186/s12933-016-0365-x (PMC4818874; doi:10.1186/s12933-016-0365-x)
Supplement: Supplementary file 1 — 10.1186/s12933-016-0365-x The drugs analyzed are listed as following. [file 12933_2016_365_MOESM1_ESM.doc]

**Supplement**

**The drugs analyzed are listed as following.**

**Insulins**:

insulin aspart;

insulin detemir;

insulin glargine;

insulin human;

insulin lispro;

**Thiazolidinediones:**

pioglitazone;

rosiglitazone;

**Sulfonylurea:**

chlorpropamide;

glibenclamide;

glibornuride;

gliclazide;

glimepiride;

glipizide;

gliquidone;

tolazamide

tolbutamide

**Dipeptidyl peptidase-4 inhibitors:**

linagliptin;

sitagliptin;

saxagliptin

vildagliptin

**Glucosidase inhibitors**

acarbose

**angiotensin-converting enzyme inhibitors**

benazepril

benazepril + amlodipine

captopril

captopril + hydrochlorothiazide

cilazapril

enalapril

enalapril + hydrochlorothiazide

fosinopril

imidapril

lisinopril

perindopril

perindopril + indapamide

quinapril

ramipril

ramipril + felodipine

**angiotensin II receptor blockers**

candesartan

candesartan + hydrochlorothiazide

eprosartan

irbesartan + hydrochlorothiazide

losartan + hyrochlorothiazide

olmesartan

olmesartan + amlodipine

olmesartan + hydrochlorothiazide

olmesartan + amlodipine + hydrochlorothiazide

telmisartan

telmisartan + amlodipine

telmisartan + amlodipine + hydrochlorothiazide

valsartan

valsartan + amlodipine

valsartan + amlodipine + hydrochlorothiazide
